# Enhancing the Cognitive Effects of Flavonoids With Physical Activity: Is There a Case for the Gut Microbiome?

**DOI:** 10.3389/fnins.2022.833202

**Published:** 2022-02-22

**Authors:** Carol L. Cheatham, David C. Nieman, Andrew P. Neilson, Mary Ann Lila

**Affiliations:** ^1^Department of Psychology and Neuroscience, University of North Carolina at Chapel Hill, Chapel Hill, NC, United States; ^2^Human Performance Lab, Department of Biology, Appalachian State University, Kannapolis, NC, United States; ^3^Department of Food, Bioprocessing and Nutrition Sciences, Plants for Human Health Institute, North Carolina State University, Kannapolis, NC, United States

**Keywords:** age-related, cognition, neuroprotective, phenolic metabolites, prebiotic flavonoids, exercise, microbiome

## Abstract

Age-related cognitive changes can be the first indication of the progression to dementias, such as Alzheimer’s disease. These changes may be driven by a complex interaction of factors including diet, activity levels, genetics, and environment. Here we review the evidence supporting relationships between flavonoids, physical activity, and brain function. Recent *in vivo* experiments and human clinical trials have shown that flavonoid-rich foods can inhibit neuroinflammation and enhance cognitive performance. Improved cognition has also been correlated with a physically active lifestyle, and with the functionality and diversity of the gut microbiome. The great majority (+ 90%) of dietary flavonoids are biotransformed into phytoactive phenolic metabolites at the gut microbiome level prior to absorption, and these prebiotic flavonoids modulate microbiota profiles and diversity. Health-relevant outcomes from flavonoid ingestion may *only* be realized in the presence of a robust microbiome. Moderate-to-vigorous physical activity (MVPA) accelerates the catabolism and uptake of these gut-derived anti-inflammatory and immunomodulatory metabolites into circulation. The gut microbiome exerts a profound influence on cognitive function; moderate exercise and flavonoid intake influence cognitive benefits; and exercise and flavonoid intake influence the microbiome. We conclude that there is a potential for *combined* impacts of flavonoid intake and physical exertion on cognitive function, as modulated by the gut microbiome, and that the combination of a flavonoid-rich diet and routine aerobic exercise may *potentiate* cognitive benefits and reduce cognitive decline in an aging population, via mechanisms mediated by the gut microbiome. Mechanistic animal studies and human clinical interventions are needed to further explore this hypothesis.

## Introduction

The brain can be a neglected aspect of human health. Typically, brain health is not a consideration until people reach their 50s or 60s, when brain function can become less reliable. Moreover, lifestyle choices are hardly ever considered as detrimental to brain health in an otherwise healthy adult. Importantly, the number of Americans aged 65 and older is projected to nearly double from 52 million in 2018 to 95 million by 2060 (United States Census Bureau, 2021). As the life expectancy of the world’s population increases, dementia (age-related decline from previously attained cognitive levels) is a looming threat to the individual’s healthspan, and causes a heavy economic burden to families and to society. Researchers have put considerable effort into developing interventions for those who have been diagnosed with a dementia (e.g., Alzheimer’s disease, Parkinson’s disease, and Dementia with Lewy Bodies). We posit that validating proactive strategies to prevent cognitive dysfunction in the aging demographic should be a high priority.

In this review, we will explore the case for flavonoids as a prophylactic against declining brain health, investigate the modulating role(s) of the gut microbiome, and consider evidence for the potentiating influence of moderate–to-vigorous physical activity (MVPA) combined with flavonoid intake. Improved cognition is associated with a physically active lifestyle, a healthy diet, and a robust, diverse gut microbiome (the gut-brain axis) (Schlegel et al., 2019; Westfall and Pasinetti, 2019). We and others have demonstrated that flavonoid-rich food interventions (such as cocoa, berries, or tea) can attenuate biomarkers of inflammation including neuroinflammation, effectively mitigate cognitive dysfunction and decline, and sharpen cognitive function (Macready et al., 2009; Whyte et al., 2020). Dietary flavonoids act as prebiotics; as such, they can alter the profiles and the diversity of the gut microbiome, and exercise accelerates the circulation and transport of flavonoid metabolites after gut microbiome catabolism. Here we review the extant literature and weave the three – flavonoids, exercise, and the gut microbiome – together to form a more complete picture of how lifestyle affects cognition and how we can prevent the deterioration of cognitive abilities across the lifespan.

## Flavonoids and Brain Health

Flavonoids, a ubiquitous group of plant secondary metabolites with a 15-carbon structure (two phenyl rings and a heterocyclic ring; C6-C3-C6) are an indispensable component of traditional medicines, current nutraceuticals, and functional foods. Dietary flavonoids are found in tea, berryfruit, citrus and other fruit and legumes, although it is estimated that consumption in the United States falls well below dietary guidance (U.S. Department of Health and Human Services and U.S. Department of Agriculture, 2015). Over a decade ago, the potential for flavonoids to attenuate neurodegeneration was recognized (Spencer, 2009), and growing epidemiological, *in vivo*, and clinical evidence suggests that supplementation with flavonoid-rich foods benefits cognitive function (Letenneur et al., 2007; Dodd et al., 2019; Rajaram et al., 2019; Westfall and Pasinetti, 2019; Ruotolo et al., 2020; Whyte et al., 2020). In part, the underlying mechanisms may include flavonoids’ anti-inflammatory capacity and influences on endothelial function and peripheral blood flow (Amin et al., 2015; Morais et al., 2016; Warner et al., 2017; Rajaram et al., 2019). We and others have established that dietary flavonoid metabolites pass the blood brain barrier and can be localized in brain tissues (Janle et al., 2010a,b; Strathearn et al., 2014; Docampo et al., 2017; Angelino et al., 2019; Westfall and Pasinetti, 2019). Importantly, the flavonoid metabolites have been shown to deposit in brain regions that underlie learning and memory, specifically the hippocampus (Sokolov et al., 2013; Flanagan et al., 2018). Flavonoids are able to exert neuroprotective activity (even at the relatively low concentrations that reach the brain) by virtue of their ability to modulate protein and lipid kinase signaling pathways, and by inhibiting neuroinflammation, rather than merely through antioxidant activity. Absorbed flavonoids and their metabolites from foods (cocoa, berry, and tea) appear to directly interact with cellular and molecular targets (e.g., ERK and PI3-kinase/Akt signaling pathways) to improve neuronal connectivity and increase expression of neuromodulatory proteins (Williams and Spencer, 2012). Mechanistically, dietary flavonoid consumption promotes peripheral and cerebral vascular flow as well as neuronal survival and differentiation. Mounting evidence supports an association between flavonoid-rich plant-based diets and improved domains of cognition in aging, notably in executive functions, which are higher-order cognitive abilities subserved by the prefrontal lobe (Rajaram et al., 2019). These detriments in executive function begin to develop in midlife.

Preclinical and clinical studies with flavonoid-rich foods indicate that higher levels of flavonoid intake are related to improved cognitive performance and tempered cognitive decline (Macready et al., 2009; Hein et al., 2019; Whyte et al., 2020). Cocoa flavanol intake, for example, has been linked to greater brain oxygenation, and higher performance during cognitive challenge (Gratton et al., 2020), with apparent dose-dependent improvements in working memory, attention and processing speed (Socci et al., 2017). A systematic review concluded that green tea intake has a positive influence on cognition through the combined influence of green tea extract constituents, including flavonoids, L-theanine, and caffeine (Mancini et al., 2017). In preclinical experiments, however, it was administration of the green tea catechins (primarily EGCG) that were credited with improved spatial cognition learning ability in rats (improved reference and working memory) (Haque et al., 2006). Berries (a dietary resource with a highly diversified flavonoid profile), have been the intervention of choice for several trials on flavonoids and cognition (Galli et al., 2006; McGuire et al., 2006; Lau et al., 2007; Duffy et al., 2008; Williams et al., 2008; Small et al., 2014; Shukitt-Hale et al., 2015; Miller et al., 2018; Dodd et al., 2019; Whyte et al., 2020). Human clinical interventions with flavonoid subgroups anthocyanins, flavanol and flavanone over the past several years indicate potential to limit or reverse age-related declines in cognition (Ahles et al., 2021; Bird et al., 2021; Gardener et al., 2021). Blueberry-supplemented rats demonstrated elevated hippocampal levels of cAMP-response element-binding protein and extracellular signal-related kinase, and brain-derived neurotrophic factor (BDNF) compared to age-matched controls. Alteration of these signaling proteins led to better performance on a spatial working memory task (Williams et al., 2008; Vauzour et al., 2021). BDNF, known for differentiation and survival of neurons of the CNS, plays a crucial role in delay of cognitive aging by improving hippocampal plasticity, long-term memory, and neurogenesis (Cunha et al., 2010). Recently, clinical results showed that even a single acute dose of a flavonoid-rich blueberry beverage (equivalent to 200 g fresh berries; recognized as a reasonable, achievable dose) attenuated a decrease in plasma concentration of BDNF, whereas BDNF levels dropped in the placebo group (Dodd et al., 2019). Other gut-derived neuropeptides (GLP-1, GLP-2, glucagon, etc.) affect brain activity, and can be modulated by flavonoid consumption (Badshah et al., 2013; Gu et al., 2014; Kashiwabara et al., 2016; Cremonini et al., 2021), suggesting another mechanism through which flavonoids enhance brain function.

The protective effects of flavonoid consumption occur primarily in the hippocampus, a brain area critical for memory function. A 3-month intervention with blueberry extract in non-impaired older adults showed significant improvements in delayed recognition and repetition errors (Whyte et al., 2018). Krikorian et al. (2010) investigated blueberry supplementation in older adults with mild cognitive impairment (MCI), finding robust improvement in a verbal paired-associate learning test. In a trial on working memory (WM), a significant increase in signaling in the left inferior parietal gyrus and left pre-central gyrus (enhanced neural activation) was found in older adults consuming blueberries, although behaviorally, performance on the WM task improved only marginally (*p* = 0.08) (Boespflug et al., 2018). More recently, a 24-week combined blueberry and/or fish oil intervention in older adults with cognitive deficits concluded that blueberry intervention improved cognitive efficiency for everyday life activities and resilience against extraneous disturbances during recognition memory tasks (McNamara et al., 2018).

**FIGURE 1:**
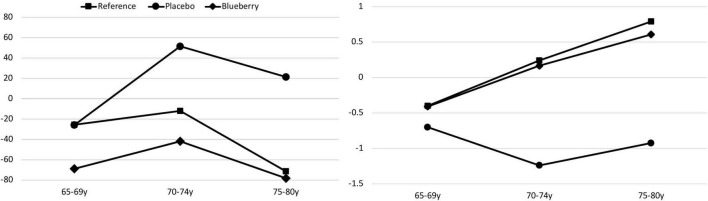
Change (delta) in speed of processing by age group across the 6-month intervention in the CANTAB rapid visual processing task (**left frame**; negative is better, *F*(2,110) = 4.14, *p* < 0.05). Recognition memory (**right frame**; positive is better) as tested by ERP (delta across 6 months) improved in the blueberry group to the level of the reference group, whereas the placebo group did significantly worse [Group: *F*(8,83) = 2.63, *p* = 0.01].

In one of the longest duration blueberry interventions to date (Cheatham et al., 2022), we randomized older adults (aged 65–80) who were experiencing age-related cognitive changes to 6 months of wild blueberry or placebo. Participants who were not experiencing cognitive changes were included as a reference group. Participants were tested for cognitive abilities using the Montreal Cognitive Assessment (MoCA) (Nasreddine et al., 2005). Age-related cognitive change was operationalized as 1–1.5 SD below the standardized mean. Participants with lower scores (>1.5 SD below the mean) were excluded from participation and referred to their physician. Cognitive abilities were gauged using the Cambridge Neuropsychological Test Automated Battery (CANTAB) and an electrophysiological technique known as event-related potentials (ERP). Those who consumed 35 g lyophilized blueberry powder/day (equivalent to ∼300 g or 2 cups fresh fruit) *did not experience any further decline* in abilities, whereas those on placebo *did*. In addition, those who consumed blueberries daily exhibited improvement in speed of processing (a basic cognitive ability that underlies all other cognitive abilities) (Figure 1, left). This improvement was evidenced in the behavioral tests (CANTAB) as well as the electrophysiological tests (ERP). In addition, recognition memory improved to the level of the reference group in the group consuming blueberries (Figure 1, right). That is, as measured in the ERP component N2, those consuming blueberries showed greater differentiation between processing (N2) in response to novel versus familiar stimuli relative to those consuming placebo. Thus, consumption of wild blueberries halted cognitive decline and improved speed of processing and recognition memory (Cheatham et al., 2022).

In sum, consumption of foods containing flavonoids appears to act in the brain to improve function (Letenneur et al., 2007; Ayaz et al., 2019). Flavonoids may enhance brain blood flow and block beta-amyloid plaque buildup (a hallmark of Alzheimer’s disease) in the brain. As prevention is generally preferred and more readily achieved than remediation, we propose that consumption of flavonoid foods are central to the prevention of cognitive decline and quite possibly other brain functions such as executive function, attention, and memory (Socci et al., 2017; Westfall and Pasinetti, 2019).

## Gut Microbiome and Cognition

The gut microbiome, an interactive community of microorganisms in the gastrointestinal tract, is highly influenced by diet (Westfall and Pasinetti, 2019; Zmora et al., 2019). Until very recently, only loosely attributed theories about the gastrointestinal microbial community’s impact on brain function and behavior were available, based on highly controlled animal studies. Recent trials, however, have shown that institutionalized study participants experiencing cognitive decline have altered gut bacterial composition compared to participants with typical cognitive ability (Scheperjans et al., 2015; Bajaj et al., 2016). These studies imply that the composition and diversity of gut microbiota may significantly modulate gut-brain communication, contributing to changes in cognition during aging (Anderson et al., 2017; Manderino et al., 2017). It has even been suggested that “westernization” of lifestyles, including western diets and the habitual use of antibiotic treatments which disrupt the gut microbiome, may contribute to neurological dysfunction (Novotny et al., 2019).

A comparative review of almost a score of recent human clinical trials suggested that deliberate intervention to change gut microbiota composition can produce a positive bacteria-cognition relationship, eliciting improvements in visuospatial memory, verbal learning, and attention (Tooley, 2020). In this narrative review, the importance of recognizing microbiota signatures associated with cognitive performance, and identifying potential gut microbiota interventions (including diet and lifestyle) were highlighted. In another clinical trial, an increased prevalence of *Bacteroides* in the gut microbiome was associated with mild cognitive impairment in geriatric patients, evidenced by impaired memory and lower global cognitive function scores (Saji et al., 2019). The gut microbiota-brain axis has previously been implicated in development of neurological diseases including Alzheimer’s disease, and is proposed as a target for cognitive decline therapeutics (Borsom et al., 2020). Deliberate modulation of gut microbiome profiles, either by fecal transplantation or probiotic interventions, is gaining research momentum in the quest to control the pathogenesis of Alzheimer’s disease (Wang and Dykes, 2021).

The microbiota-gut-brain communication is bidirectional; changes in the composition of the gut microbiota are associated with behavioral and cognitive alternations, and, perturbations in behavior also alter the composition of the gut microbiota (Mu et al., 2016; Borsom et al., 2020). Gut microbial metabolites (including postbiotics such as bile acids, short chain fatty acids, and tryptophan metabolites, as well as phenolic metabolites from flavonoid catabolism) are major mediators of the microbiome-gut-brain axis (Cryan and Dinan, 2012; Banfi et al., 2021). We posit that cognition (memory and executive function) are linked to the composition, functionality, and diversity of the gut microbiota, in part because gut microbiota *regulate the production and delivery of microbiome-catabolized phenolic metabolites into circulation following intake of flavonoid-rich foods* (Dinan et al., 2015; Manderino et al., 2017; Chu et al., 2019).

**FIGURE 2:**
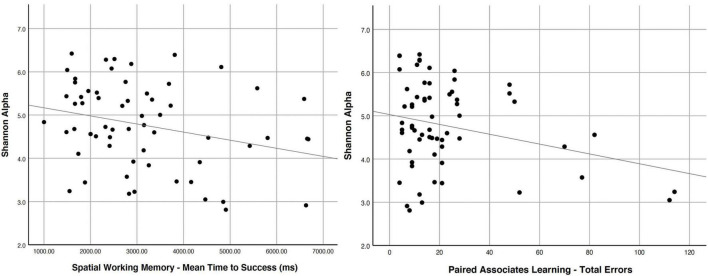
Cognitive scores (CANTAB) related to the gut microbiome diversity score Shannon Alpha [Multivariate Model *F*(2,56) = 4.846, *R*^2^ = 0.117] on spatial working memory (**left**–longer times to success relate to lower diversity; *p* = 0.025) and paired associates learning (**right**-higher errors on the task relate to lower diversity; *p* = 0.024). Reprinted from Canipe et al. (2021).

Using clinically validated behavioral measures and electrophysiological measures of brain activity on a cohort of older adults between 67 and 83 years of age and a diversity metric for the gut microbiome, we recently found that gut microbial diversity, as modulated by diet/flavonoid intake, was a predictor of cognitive performance in free-living aging adults (Canipe et al., 2021). We saw a significant association between behavioral measures of paired-associate learning and spatial working memory, and the α-diversity of the gut microbiome of older adults; poorer performance (indicative of cognitive dysfunction) predicted lower gut microbiome diversity (Canipe et al., 2021). Poorer performance on spatial working memory tests and paired associates learning was related to lower Shannon α-scores (a diversity metric) (Peet, 1974) in the gut microbiome (Figure 2). Electrophysiology waveforms related to attention differentially predicted gut microbiome diversity, such that those with better attention allocation and better sustained attention abilities had more diverse gut microbiomes. Thus, higher gut microbiome diversity is related to better brain function as measured by cognitive tests. Importantly, in this same sample, we related free-living consumption of berries to the diversity score and found that those who reported eating more servings of berries across three 24-h diet recalls had a more diverse gut microbiome) (Canipe et al., 2021). Interacting ingested flavonoids can serve a pivotal role in changing or reshaping the gut microbiota, increasing populations of *Lactobacilli* spp. and *Bifidobacteria* spp. and inhibiting gut pathogens (Wang et al., 2021). Reduced abundance of pathogenic bacteria in the gut (*Clostridium perfringens*, *C. difficile*, and gram-negative *Bacteroides* spp.) without inhibition of commensal bacteria (clostridia and lactobacilli) has been linked to prebiotic polyphenol intake (Lee et al., 2006; Tuohy et al., 2012; Duda-Chodak et al., 2015). Separate *in vivo* feeding trials with berry species (cranberry and grape) led to consistent decrease in proportion of Firmicutes to Bacteroidetes, and remarkable increase in growth of *Akkermansia muciniphila* in the microbial community (Anhe et al., 2014; Roopchand et al., 2015; Wang et al., 2021).

In sum, consumption of flavonoids is related to improved speed of processing and recognition memory; consumption of berry flavonoids is related to a more highly diverse gut microbiome; and higher α-diversity in the gut microbiome is related to better cognitive abilities (Canipe et al., 2021). The next section will further explore how flavonoid consumption alters the gut microbiome.

## Flavonoids and the Gut Microbiome

Interactions between the microbiome and dietary flavonoids have dual impacts on human health. First, diet, including prebiotic flavonoid-rich foods, has a dramatic influence on the composition and consequently the functionality of the gut microbiome. The term prebiotic usually refers to indigestible dietary fibers that benefit the gut microbiota, and flavonoids are considered to have prebiotic-like effects as their consumption also feeds/benefits commensal bacteria in the gut. Ingesting prebiotics alters gut microbial community structure, favoring beneficial commensal bacteria, and reducing levels of opportunistic species (Anhe et al., 2014; Roopchand et al., 2015; Westfall et al., 2018). Prebiotic flavonoids’ metabolites also have a profound influence on inflammation in the gut, improving the epithelial barrier’s integrity and activating tight junctions (Anhe et al., 2014; Westfall and Pasinetti, 2019). Interestingly, the flavonoids do not even need to be absorbed to exert these benefits; as xenobiotic compounds, they induce cellular stress and an overcompensation reaction to maintain homeostasis, producing a hormetic response that improves cell and barrier function (Calabrese et al., 2012). The same gut-derived flavonoid metabolites that suppress chronic intestinal inflammation are integral to inhibition of neuroinflammation (Spencer et al., 2012).

In tandem, the gut microbiome significantly impacts flavonoid bioavailability via extensive pre-systemic metabolism to release active metabolites with therapeutic efficacy, including for cognitive benefits. Only a small percentage of dietary flavonoids are absorbed from the small intestine, before they reach the colonic microbiota. Instead, most dietary flavonoids are biotransformed by commensal gut microbiota into diverse bioactive phenolic metabolites, and are delivered into circulation where they elicit health-protective effects (Jennings et al., 2019). *The bioavailability of flavonoids is therefore largely dependent on their catabolism by the gut microbiome and subsequent secondary xenobiotic biotransformation in the liver before entering circulation in the form of phenolic metabolites* (Espin et al., 2017; Williamson et al., 2018; Westfall and Pasinetti, 2019). Thus, flavonoid bioavailability depends, in part, on the polyphenol-microbiota interactions, which ultimately regulate both bioavailability and bioactivity (Lila et al., 2016).

Extensive evidence indicates that biotransformed metabolites from lower-intestinal bacterial catabolism of dietary flavonoids mediate anti-inflammatory activity in multiple tissues of the body (Moco et al., 2012; Morais et al., 2016; Tomas-Barberan et al., 2016; Schell et al., 2019; Deledda et al., 2021; Martin and Ramos, 2021). Notably, diseases which cause chronic low-grade inflammation (metabolic syndrome, diabetes, and arthritis) are strongly linked to cognitive decline in aging (Santoro et al., 2014; Noble et al., 2017). Strategies to attenuate the chronic, low grade inflammatory status characteristic of aging adults (inflammaging) can evoke systemic benefits on both physical and cognitive health. The gut microbiota is also essential for producing the full battery of bioavailable plasma- and brain-bioactive metabolites that have neuroprotective capacity (Westfall and Pasinetti, 2019). Long-term supplementation with a probiotic could promote health by introducing colonic microbiota that make flavonoids more bioavailable (Pereira-Caro et al., 2015; Westfall and Pasinetti, 2019).

Evidence from our team and others shows that gut microbial-derived metabolites of dietary flavonoids are anti-inflammatory and immunomodulatory, can have greater bioactivity than their parent/precursor structures, and have additive or synergistic effects collectively (Ahmed et al., 2014; Amin et al., 2015; Duda-Chodak et al., 2015; Nieman et al., 2017, 2018; Warner et al., 2017). Indeed, daily consumption of anthocyanins in a diet-induced obese mouse model with either healthy or antibiotic-disrupted gut microbiota resulted in reduced body weight gain and improved glucose metabolism, but only in mice with intact gut microbiota (Esposito et al., 2015). The bidirectional breakdown of flavonoids into active and more bioaccessible metabolites and concurrent modulation of the gut microbial community by these metabolites, both contribute to positive health outcomes.

So far, we have described the positive associations between flavonoid ingestion and cognitive health; the connections between a robust microbiome and cognitive health; and the two-way interactions between the gut microbiome and ingested flavonoids, which mediate both the potency and bioavailability of flavonoid metabolites; and the composition and functionality of the microbiome. How might lifestyle factors, aside from diet, have a bearing on these cognition-relevant influences?

## Physical Activity as a Mediator

A physically active lifestyle is intrinsically linked to brain health. Physically active people are less likely to demonstrate cognitive decline, all-cause dementia, vascular dementia, and Alzheimer’s disease; most data support MVPA for at least 150 min per week (Middleton et al., 2008; Voss et al., 2011; Gow et al., 2012; Napoli et al., 2014; Piercy and Troiano, 2018; Minghui, 2019; Chen et al., 2020). MVPA for elderly adults has benefits for cognitive performance, brain function, and brain structure (Voss et al., 2011). Most meta-analyses and systematic reviews support that regular MVPA improves various aspects of cognitive function including executive function (EF), language ability, visuospatial ability, and memory in older adults with cognitive impairment (Chen et al., 2020; Zhou et al., 2020). When physical activity was directly monitored, participants engaging in recommended levels of MVPA had lower incidence of cognitive impairment and better maintenance of EF and memory (Zhu et al., 2016).

The Physical Activity Guidelines for Americans state that some benefits of physical activity on cognitive health occur immediately after a session of MVPA (acute effect); these include reduced anxiety, improved sleep, and improved cognitive function (Piercy and Troiano, 2018). With regular MVPA by older adults with or without impaired cognitive health (chronic effect), even greater cognitive benefits are experienced including improvements in EF, attention, memory, crystallized intelligence, and processing speed (Weng et al., 2015; Tsukamoto et al., 2017; Piercy and Troiano, 2018). The substantial cognitive benefits observed with at least 150 min per week MVPA are further amplified when MVPA is increased to 300 min per week. Potential mechanisms for MVPA’s positive impacts on cognition include changes in brain structures (Rovio et al., 2010; Devenney et al., 2017), increases in cerebral blood flow and oxygentation (Rooks et al., 2010; Tsubaki et al., 2021), enhanced immune function, reductions in inflammation including neuroinflammation, and/or increase neurotrophic factors (Nieman et al., 2013; Ahmed et al., 2014; Amin et al., 2015; Rehfeld et al., 2018; Minghui, 2019). Habitual walking in late adulthood has been correlated with higher gray matter volume, coincident with reduced risk of cognitive dysfunction (Erickson et al., 2010). Flavonoid benefits coincident with physical exertion are in part due to antioxidant and anti-inflammatory effects, but also since these polyphenols activate the same adaptive cell signaling pathways as physical exertion, they are believed to complement adaptive benefits of exercise and support performance (Hurst et al., 2019).

Moderate-to-vigorous physical activity combined with flavonoid ingestion may improve post-exercise metabolic recovery (Hurst et al., 2019; Nieman et al., 2019, 2020), and augment cognitive function (Tsukamoto et al., 2018). Effects of MVPA and flavonoid ingestion may be mediated in part through elevations in circulating gut-derived phenolic metabolites, but this linkage has not yet been conclusively established (Nieman et al., 2013, 2018). MVPA does enhance the release of gut-derived phenolic metabolites following chronic flavonoid ingestion. In a randomized trial with long-distance runners featuring a 17-day intervention with a flavonoid-rich supplement (or placebo), serum metabolic signatures from colonic flavonoid metabolites (derived from green tea or berries) were significantly elevated for at least 14 h coincident with a 3-day intensified exercise period, and these changes persisted through post-exercise recovery (Nieman et al., 2013). Microbial metabolites were dramatically elevated once the workout commenced – the release of the metabolites into plasma was stimulated by physical exertion. Release of these microbial metabolites into circulation significantly countered the athletes’ typical post-exercise susceptibility to virus infection, by depressing *ex vivo* viral replication and attenuating virulence (Ahmed et al., 2014).

**FIGURE 3:**
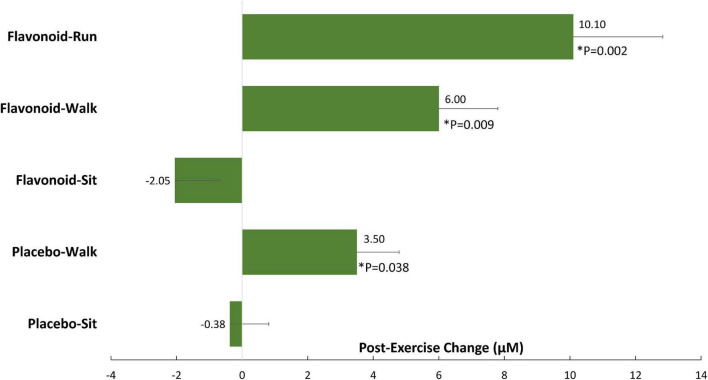
Post-exercise change for 15 selected and grouped plasma gut-derived phenolics. *P*-values indicate contrasts with the placebo–sit group. Data are represented as means with standard errors of the mean. *indicates a significant difference with the placebo-sit group (*P* values).

In another study, the combination of 2 weeks of flavonoid supplementation and acute exercise (both 45 min brisk walking and 2.5 h running) enhanced the translocation of gut-derived phenolics into circulation (Figure 3; Nieman et al., 2018). The pre-study plasma concentration of gut-derived phenolic metabolites was 40% higher in the leaner and fitter runners than in the walkers. These data indicated that acute exercise bouts (both brisk walking and intensive running) combined with flavonoid supplementation, and an elevated fitness status associated with habitual running, were linked to elevations in plasma levels of gut-derived phenolics.

Several underlying mechanisms could explain these results including flavonoid- and exercise-induced changes in gut permeability and transporter function, increases in gut microbiota richness, and altered gastrointestinal motility and transport rate (Nieman et al., 2018). Chronic MVPA can modify the composition and functional capacity of the gut microbiota (Hughes and Holscher, 2021). Cross-sectional human studies have revealed greater α-diversity and an enriched profile of short chain fatty acids (SCFAs) in athletes compared to sedentary controls (Barton et al., 2018; Mailing et al., 2019; Nieman and Pence, 2020; Zhu et al., 2020). One examination of phenotypic features across 3,400 individuals found a linkage between microbiome diversity and MVPA frequency and duration that was independent of major dietary factors and BMI (Manor et al., 2020) and longitudinal MVPA studies support some selective changes on the gut microbiome, especially when vigorous exercise training is sustained for months (Allen et al., 2018; Cronin et al., 2018; Kern et al., 2020). The interaction between MVPA and the gut microbiota is bidirectional. As already noted, flavonoids are catabolized by and influence composition of the microbiota, and exercise stimulated the bioavailability of flavonoid metabolites (Nieman et al., 2018). The gut microbiota also has an influence on exercise performance by producing SCFAs that increase muscle blood flow and insulin sensitivity, and can be utilized as fuel (Hughes and Holscher, 2021).

**FIGURE 4:**
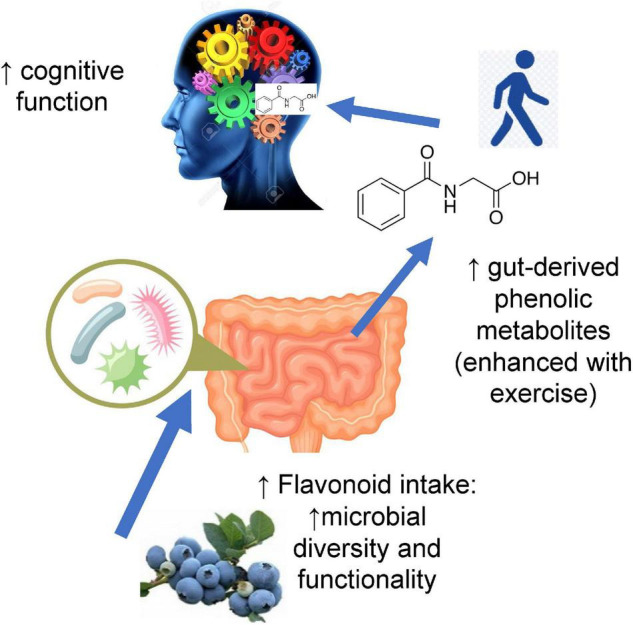
Higher flavonoid intake may influence cognitive function by augmenting gut microbial diversity and functionality and increasing circulating levels of gut-derived phenolic metabolites. Moderate-to-vigorous physical activity (MVPA) adds to this effect by enhancing the release of gut-derived metabolites and improving cognitive function.

It is therefore reasonable to posit that the connection between physical activity (e.g., exercise), dietary flavonoids, and improved cognition (memory and executive function) is linked to their demonstrated influence on the microbiome (diversity and functionality) (Figure 4).

## Conclusion

A complex interaction of factors (lifestyle, diet, genetics, and environment) all appear to exert influence on cognition in the aging brain. Emerging evidence suggests that there may be *potentiating interactions* between some of these, including flavonoid intake, microbiome, and physical activity levels (exercise). What are the mechanisms responsible for the benefits to cognition, and how can they be fully demonstrated? We hypothesize that it is the phytoactive metabolites from flavonoid ingestion, *after catabolism at the gut microbiome level*, that interact with cellular and molecular targets (signaling pathways) to improve neuron connectivity and promote vascular and peripheral flow in the brain. Exercise has demonstrated ability to provoke a surge of these phytoactive flavonoid metabolites into circulation. It follows that the positive cognitive benefits from dietary flavonoids and regular moderate exercise may be a consequence of the enhanced circulation of gut-derived flavonoid metabolites, mediated by the activities of the colonic microbiota.

## Author Contributions

CC: cognition. DN: physical movement. AN: microbiome. ML: flavonoids/metabolites. All authors contributed to and approved the manuscript.

## Conflict of Interest

The authors declare that the research was conducted in the absence of any commercial or financial relationships that could be construed as a potential conflict of interest.

## Publisher’s Note

All claims expressed in this article are solely those of the authors and do not necessarily represent those of their affiliated organizations, or those of the publisher, the editors and the reviewers. Any product that may be evaluated in this article, or claim that may be made by its manufacturer, is not guaranteed or endorsed by the publisher.
